# Diagnosing Constipation Spectrum Disorders in a Primary Care Setting

**DOI:** 10.3390/jcm10051092

**Published:** 2021-03-05

**Authors:** Joel Heidelbaugh, Nicole Martinez de Andino, David Pineles, David M. Poppers

**Affiliations:** 1 Department of Family Medicine, University of Michigan, Ann Arbor, MI 48109, USA; jheidel@med.umich.edu; 2 Division of Gastroenterology/Hepatology, Department of Surgery, Medical College of Georgia, Augusta University, Augusta, GA 30912, USA; nmartinezdeandino@augusta.edu; 3 Division of Gastroenterology, Department of Medicine, NYU Langone Health, New York, NY 10022, USA; David.Pineles@nyulangone.org; 4 Center for Advanced Therapeutics and Innovation, NYU Langone Health, New York, NY 10022, USA

**Keywords:** chronic idiopathic constipation, constipation, irritable bowel syndrome, pathophysiology, primary care

## Abstract

Understanding pathophysiological causes of constipation is worthwhile in directing therapy and improving symptoms. This review aims to identify and fill gaps in the understanding of the pathophysiology of constipation, understand its prevalence, review diagnostic tools available to primary care physicians (PCPs), and highlight patients’ expectations for the management of this common spectrum of disorders. Literature searches conducted via PubMed included terms related to constipation, diagnosis, and patient perceptions. Case studies were developed to highlight the differences between patients who may be appropriately managed in the primary care setting and those requiring specialty consultation. Myriad pathophysiological factors may contribute to constipation, including stool consistency, altered intestinal motility, gut microbiome, anorectal abnormalities, as well as behavioral and psychological factors. Common diagnoses of “primary constipation” include slow-transit constipation, defecation disorders, irritable bowel syndrome with constipation, and chronic idiopathic constipation. A detailed medical history should be conducted to exclude alarm features and PCPs should be familiar with pathophysiological factors that cause constipation, available diagnostic tools, alarm signs, and the various classification criteria for constipation subtypes in order to diagnose and treat patients accordingly. PCPs should understand when a referral to a gastroenterologist, anorectal specialist, pelvic floor physical therapist, and/or mental health specialist is appropriate.

## 1. Introduction

Chronic constipation affects up to 14% of people during their lifetime [1]. Therefore, primary care physicians (PCPs) need to be familiar with constipation subtypes and their diagnosis. PCPs are equipped to manage constipation and should be confident in determining the cause of their patient’s constipation [2] to ensure that appropriate treatment options are considered and recommended. Finally, PCPs should be aware when subspecialty referral is advised, such as when worrisome features (“red-flags”) are present.

**Figure 1:**
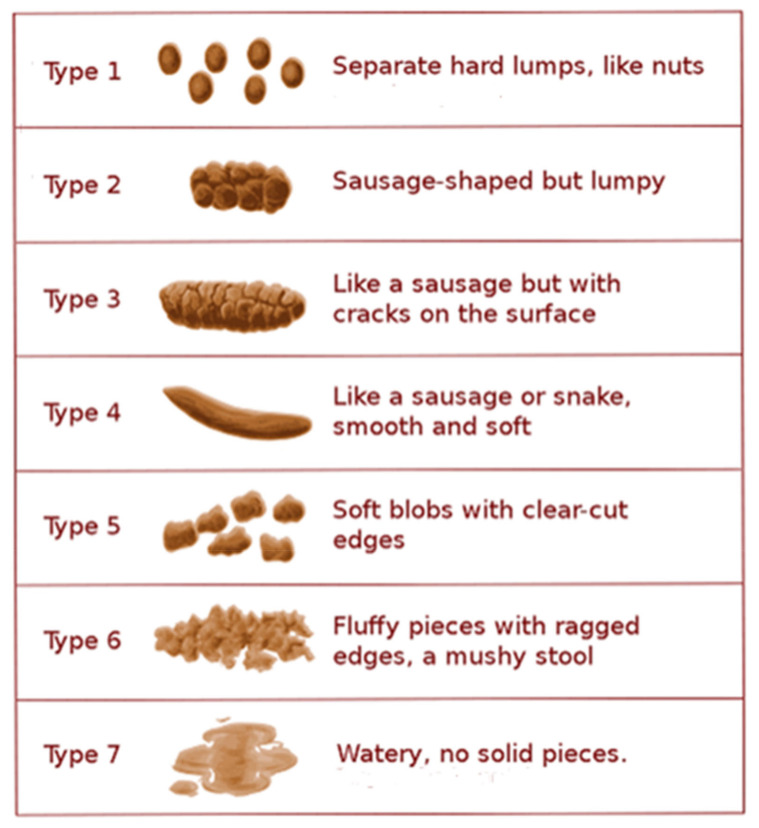
Bristol Stool Form Scale. Copyright 2000 © by Rome Foundation. All Rights Reserved.

Chronic constipation is a gastrointestinal disorder that is characterized by lumpy or hard stools, infrequent bowel movements, abdominal cramping, bloating, excessive straining, and/or the sensation of incomplete defecation or “evacuation” [3]. The Rome IV criteria provide a straight-forward guide for diagnosing chronic constipation [4]. A key tool that can be used to aid in diagnosis of constipation is the Bristol Stool Form Scale (BSFS; Figure 1), which categorizes stool form on a graded 7-point scale ranging from separate hard lumps that are difficult to evacuate (BSFS type 1) to mushy, watery stools (BSFS type 7) [5]. Amongst others, the criteria for constipation include BSFS type 1 or type 2 stools in over 25% of bowel movements and less than 25% of BSFS type 6 or type 7 stools [4]. Patients may also provide self-diagnoses based on their complaints (i.e., hard stools or straining) or by the use of laxatives to relieve symptoms, and it is worthwhile to note that there are discrepancies in the perception of constipation between primary physicians and the patients whom they treat [6].

Possible causes of constipation are myriad and include insufficient liquid and/or fiber intake, abnormalities in colonic motility, reduced exercise, and physical disorders (e.g., neuromuscular disorders) [7]. Although insufficient liquid intake or limited exercise alone may not be the sole contributing factor in causing constipation, there is evidence suggesting that improved liquid intake and physical activity can improve constipation symptoms in certain patients [8]. Chronic constipation may or may not have an identifiable cause; causes may be primary (related to intrinsic gastrointestinal structure and function) or secondary (related to systemic disease or medication) [1,9]. An identifiable cause of constipation is unknown in the largest subset of chronic constipation sufferers [10]. Primary constipation can be caused by functional colonic abnormalities or by defects in the process of defecation itself [9]. Primary constipation may be present in patients with inflammatory bowel disease (IBD), which includes Crohn’s disease (CD), ulcerative colitis (UC) [11], and indeterminant colitis. IBD is more classically associated with diarrhea and bloody stools; however, IBD represents a spectrum in which altered motility and obstruction may occur. Secondary constipation often has an identifiable cause related to medication use or to other underlying disease processes [1,9]. Secondary constipation may be associated with medication side effects, bowel obstruction (including secondary to adhesions, malignancy, benign strictures, or extrinsic bowel compression from other organs or abnormal lesions within the peritoneal cavity), metabolic disorders (e.g., hypothyroidism, hypercalcemia), neurological disorders (e.g., Parkinson’s disease, multiple sclerosis), other systemic disorders (e.g., scleroderma, amyloidosis), as well as psychological disorders (e.g., depression, eating disorders) [9].

## 2. Patient’s Perspective of Constipation Spectrum Disorders

A patient’s quality of life (QoL) is understandably negatively correlated with the severity of constipation [1,3,12]. While constipation is defined by characteristics of a patient’s bowel habits, the patients underlying concerns may be unrelated (or only partially related) to their bowel movements [1]. The symptom that patients perceive as the most severe is normally the one that they feel is the most bothersome; this may include pain, bloating, abdominal discomfort, or some combination thereof [12]. Patients often report an increasing severity of their symptoms over time (i.e., the longer they have symptoms, the more severe and bothersome they perceive them to be) [12]. Patients often utilize over the counter (OTC) therapies prior to discussing their symptoms with their PCPs. They are often dissatisfied with the results of traditional first-line therapies (such as fiber and over-the-counter laxatives), potentially because these focus on symptomatic management rather than addressing the underlying *causes* of their chronic constipation [12]. It is important to discuss previous treatments with patients in detail because isolated fibers (supplements) may have varying effects on constipation; both psyllium and coarse wheat bran improved symptoms, while finely ground wheat bran may have an unwanted stool-hardening effect [13]. In addition, fiber and/or laxatives do not benefit patients with certain types of chronic constipation, such as functional defecation disorders (DDs), and in fact may exacerbate these issues in a subset of these patients [12,14].

Many patients with constipation have become accepting of the physical and QoL limitations of their symptoms [3] and may not be aware of available effective treatments. Constipation may be associated with depression, anxiety, and other psychosocial issues [12]. It is therefore recommended that PCPs explain to patients how chronic constipation may impact their QoL. In certain cases, in which significant impact upon QoL is noted, early involvement of mental health professionals may be beneficial.

In the absence of alarm symptoms, the most important role of the PCP is to consider how to best manage the expectations of patients with chronic constipation by discussing, among other issues, what tests might need to be performed to confirm (or exclude) diagnoses, the available treatment options and their likelihood of success, potential treatment-related adverse events (such as diarrhea), and options to consider if initial lines of therapy are unsuccessful or do not yield adequate relief. In many cases, empiric therapy may be recommended without diagnostic investigation; however, some limited testing may be clinically warranted depending on the individual patient (e.g., complete blood count, thyroid-stimulating hormone, complete metabolic panel, age-appropriate colorectal cancer screening) [4].

## 3. Diagnosing Constipation in a Primary Care Setting

The diagnosis and clinical presentation of constipation may also be influenced by patient factors. The prevalence of constipation can vary depending on gender, age, race, and socioeconomic status [15,16]. Women experience constipation at a rate 2.2-fold higher than that in men [17]. While a younger patient population may report increased constipation symptom severity [18], the prevalence of constipation increases with age and is much higher in patients aged ≥ 65 years [15,17]. Older patients with higher rates of polypharmacy may also be at risk for drug–drug interactions, thereby complicating treatment efficacy and safety [19,20]. Race and socioeconomic status may also be factors in the risk of developing constipation, with a higher rate of constipation reported in non-white versus white patients and in those of lower socioeconomic status [16,21].

There exist only a few widely used, validated, and standardized tools for the classification of constipation. The BSFS categorizes stool forms ranging from liquid stools to stools that are hard and lumpy in consistency (Figure 1) [5]. By asking patients to indicate their stool form on the BSFS chart, insight can be gained as to the nature of the patient’s stool and whether they are consistent with constipation. Rome IV provides criteria to aid in the diagnosis and subclassification of functional GI disorders, including CIC (i.e., functional constipation) and IBS-C (Table 1). According to Rome IV, a constipation diagnosis should be made following a clinical history, physical examination, minimal laboratory tests, and, when clinically appropriate, a colonoscopy or other diagnostic test (such as age-appropriate colorectal cancer screening) [4]. Although currently the Rome criteria may not be widely used by PCPs, it a useful tool that, when utilized in the primary care setting, would help standardize constipation diagnosis and drive appropriate treatment decisions.

Internists and other primary physicians should enquire if, and how, a patient may have already attempted to manage their constipation. On average, patients used three OTC products before consulting a health care professional [3]. The failure or inadequacy of previous therapies, and behavioral, dietary, and lifestyle modifications may provide insight into a potential diagnosis [1] as well as the next diagnostic and therapeutic steps that may be considered, potentially minimizing unnecessary delays in treatment escalation or specialist referral. Common OTC therapies for constipation include supplemental fiber, stool softeners, probiotics, prebiotics, and nonprescription laxatives, which are relatively cost-effective compared to prescription treatment [25]. However, only 40% of patients report satisfaction with OTC laxatives [3].

PCPs must capture a detailed clinical history to exclude alarm symptoms (which may indicate a more serious health problem) and evaluate for common comorbidities that may be driving secondary causes of constipation [4]. These alarm symptoms that can present with constipation include unintentional weight loss, iron-deficiency anemia, hematochezia (rectal bleeding/bloody bowel movements), new onset of symptoms at age 50 or older, and/or severe, persistent, and treatment-refractory constipation [2,26]. Patients with the above symptoms (or other “red flag” symptoms) and those with a family history of colorectal cancer, IBD, or celiac disease should also be considered for expedited specialist referral [1,2]. When taking a patient’s history, secondary constipation should be considered [9]. PCPs should be aware if their patient is taking certain medications that can cause secondary constipation [1] and if they have a history of neurologic, endocrine, and metabolic disorders, which may be associated with constipation (Table 2). Opioids alone or in combination with other medications may contribute to chronic constipation [1]. Several therapies specifically target opioid-induced constipation [27], and should be considered as part of the management strategy when opioid-induced constipation is diagnosed. Colorectal cancer screening should be pursued if the patient is not up to date with recommendations [4].

Based on the individual patients’ history, their risk factors, and the clinician’s degree of suspicion with regard to the cause of their constipation, PCPs should determine whether excluding other etiologies by objective testing, imaging, etc., is necessary. However, in the absence of alarm symptoms, if there are comorbidities potentially contributing to constipation, primary physicians can typically manage by empiric therapy and monitoring of outcomes.

**Figure 2:**
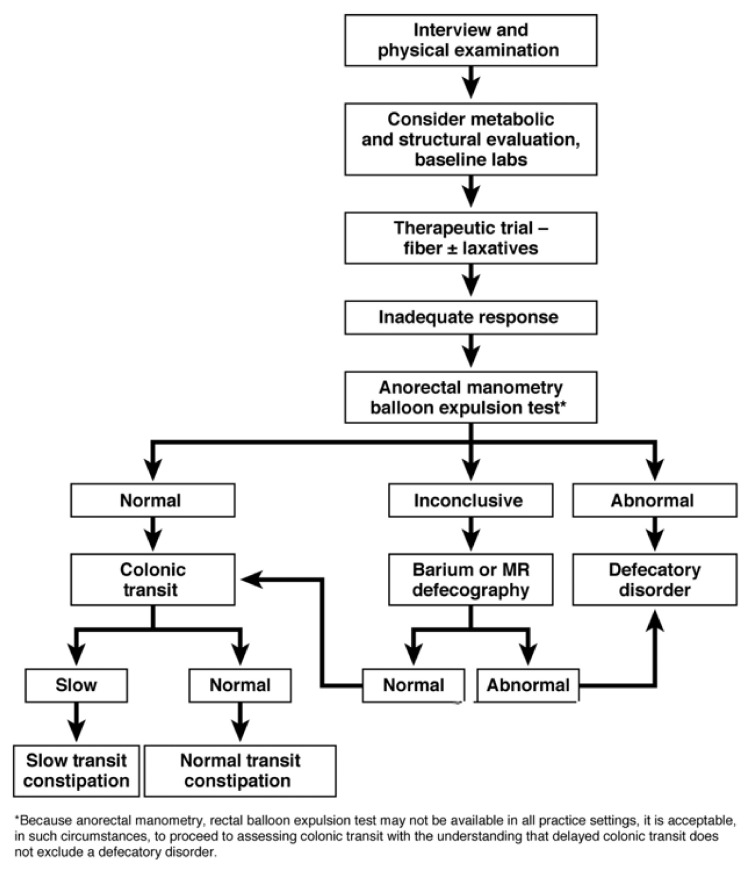
Diagnosing chronic constipation, Reprinted from *Gastroenterology*, Vol. 144, Bharucha, A.E., Dorn, S.D., Lembo, A., Pressman, A., American Gastroenterological Association Medical Position Statement on Constipation, Pages 211–217, 2013, with permission from Elsevier [28]. * As anorectal manometry/rectal balloon expulsion test may not be available in all practice settings, it is acceptable in such circumstances to proceed to assessing colonic transit with the understanding that delayed colonic transit does not exclude a defecatory disorder. MR, magnetic resonance.

Primary constipation is often a complex condition that can be challenging for primary physicians (and indeed subspecialists) to manage [22]. If constipation and its associated symptoms are severe, not improving with conservation and first-line therapies, or are of unclear etiology, the patient should likewise be considered for specialist referral (Figure 2) [2].

As part of a detailed physical exam, abdominal and digital rectal examination (with consideration of in-office anoscopic evaluation in selected patients) should be performed at the time of discussion of the patient’s presenting symptoms to help begin to elucidate the cause of chronic constipation [29]. Explaining the purpose and nature of the digital rectal examination to patients with chronic constipation is recommended, as well as the relevance of anorectal anatomy as it relates to defecation and stool evacuation. This topic should be broached sensitively, particularly in select patient populations. A digital rectal exam should be performed in the presence of chronic constipation to assess for rectal tone, puborectalis muscle function, inappropriate anal contraction during defecation, abnormal perineal descent during defecatory effort, and the suggestion of potential dyssynergic defecation; related postural and respiratory function issues may also be assessed during a physical exam [30]. Palpable abnormalities during a digital rectal exam may require further testing or examination, including imaging studies, manometric or other studies, and possibly consultation by other medical colleagues, such as gastroenterologists, specialists in anorectal disorders (including colorectal surgeons), and pelvic floor–trained physical therapists. PCPs should know how to perform a proper digital rectal examination and assess tone and perianal decent as it is often a revealing element to the clinical evaluation [29]. References are available to guide performing and interpreting digital rectal examinations [31].

### 3.1. Primary Constipation Diagnoses

Irritable bowel syndrome with constipation (IBS-C) is diagnosed using the Rome IV criteria, in which abdominal pain is required for a diagnosis [4]. Abdominal bloating is a very common feature of IBS but is not required for this diagnosis (Table 1) [2]. IBS-C is complex and can involve multiple mechanisms [2,22]. Specifically, IBS-C may be associated with alterations in motor and secretory functions of the gut [32,33], gut microbiota (“the gut microbiome”), visceral hypersensitivity or hyperalgesia, mucosal dysfunction, and immune dysfunction [22,34]. When the intestinal mucosal barrier is impaired, bacteria may be able to traverse this barrier, which can cause gastrointestinal pain and exacerbate baseline psychological disorders, such as anxiety and depression [22]. IBS is the most commonly diagnosed gastrointestinal tract disorder that is associated with psychological factors [22], although it is important to note that not all psychological conditions lead to the development of IBS. Stress can affect the brain–gut connection (a connection between the central nervous system and enteric/gut-based nervous system), resulting in abnormalities in colonic motility, such as prolonged colonic transit [22]. Impaired serotonin release has been observed in patients with IBS-C [22,35].

Chronic idiopathic constipation is the most commonly diagnosed subtype of chronic constipation [9,10] and can be divided into normal-transit constipation, slow-transit constipation (STC), and DDs [4]. CIC is diagnosed when there are no identifiable physiological or biochemical etiologies of the symptom complex [10]. According to the Rome IV criteria, CIC is diagnosed when a patient presents with chronic constipation but does not meet all the criteria for IBS-C; the main difference is that pain is not a predominant symptom or may not be present in CIC [4,10,36]. Abdominal bloating, when present, can be a challenging symptom of CIC [37]. Patients with CIC may respond well to increased dietary fiber and other conservative measures, although these measures may potentially exacerbate abdominal bloating. These patients may also require more intensive or targeted pharmacologic therapies [9]. Slow-transit constipation is characterized by infrequent bowel movements (typically fewer than once per week), decreased defecatory urge, and bloating or abdominal discomfort [9]. Patients with STC have a prolonged colonic transit time [9]. STC is thought to be caused by a neuromuscular disorder of the colon [9]. For example, patients may have a decreased number of interstitial cells of Cajal (ICCs), which help regulate contractions in the gastrointestinal tract [9]. Colonic transit time may be assessed using a variety of techniques, including radiopaque markers, wireless motility capsules, or scintigraphy [1,38].

Defecation disorders are heterogeneous in nature. Some may be characterized by excessive straining [9]; however, symptoms have a limited utility in determining a DD diagnosis [39]. Patients tend to spend large amounts of time on the toilet each day [9]. In addition, increased pelvic floor tone in patients with DD may increase their risk of hemorrhoids and anal fissure [9]. Underlying structural or mechanical abnormalities may be present [9]. Laxatives are often ineffective and patients may have difficulty evacuating liquid stools [9]. Assessing for dyssynergia includes evaluating a patient for paradoxical increases in anal contraction or decreases in resting anal sphincter pressure or inadequate propulsive forces [9]. A digital rectal exam can assess for abnormal anal contraction while straining, in which case further specialist testing may be needed to confirm a specific defecatory disorder [2]. Gastroenterologists with a focus on anorectal disorders may perform additional diagnostic tests, including anorectal manometry, balloon expulsion testing, and magnetic resonance (MR) defecography with qualified radiology colleagues, along with pelvic floor physical therapy and biofeedback in concert with those appropriately trained and experienced in this area (Figure 2) [9].

### 3.2. Overlap Between IBS-C and CIC

There is often significant overlap between IBS-C and CIC. As such, both conditions are often considered to exist on a spectrum of functional constipation disorders rather than as distinct entities, often making it challenging to distinguish in the primary care as well as specialty clinical setting (Table 1) [1,2,4,23,24,36]. Per Rome IV criteria, abdominal pain is the discriminating factor of IBS-C as compared with CIC [2,36]. Pain may be a marker of disease severity, but not necessarily a distinguishing factor in all cases per se [1,2]. Indeed, at times, an individual’s symptoms may fluctuate between those more consistent with CIC or IBS-C [2,23].

## 4. Pathophysiology

**Figure 3:**
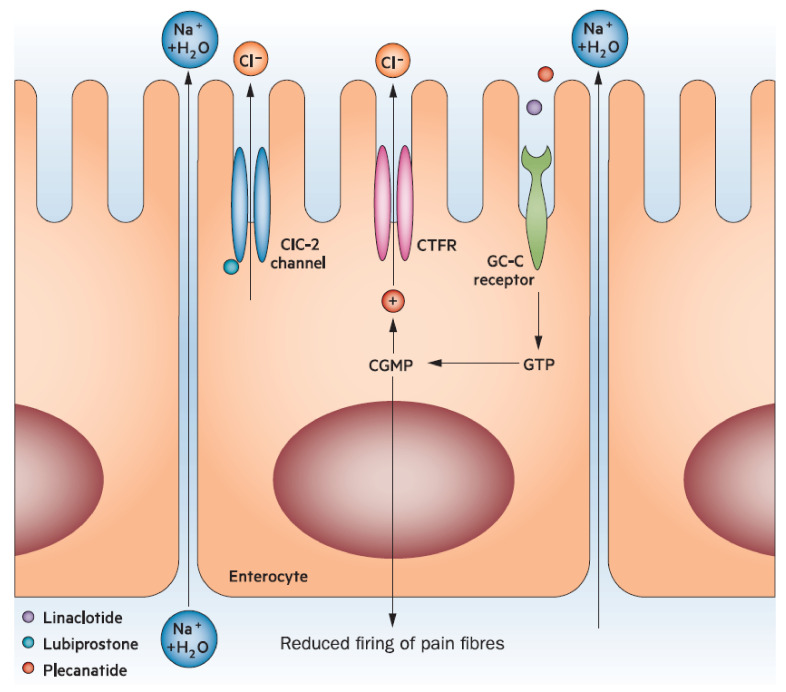
Mechanism of action of common therapies used to manage chronic constipation. Reprinted by permission from Springer Nature Custom Service Center GmbH: Nature Reviews Gastroenterology & Hepatology Agents that act luminally to treat diarrhea and constipation. Menees, S., Saad, R., Chey, W.D., 2012 [42]. Chloride channel activation in the treatment of constipation. ClC-2, type-2 chloride channel; CFTR, cystic fibrosis transmembrane regulator; GC-C, guanylate cyclase-C; GTP, guanosine triphosphate; cGMP, cyclic guanosine monophosphate.

Many pathophysiological factors can cause constipation, including abnormalities in colonic absorption, colonic motility, as well as behavioral and psychological factors [1]. Water content, and thereby stool consistency, may correlate with colonic transit time [1,9,22]. Secretion of water into the intestinal lumen is essential for normal stool consistency. The longer stools take to pass through the colon, the more water is absorbed by the colon, thereby increasing the firm consistency of stool [9]. This process may contribute to issues such as the passage of small hard stools (BSFS type 1), or large hard stools (BSFS type 2), both of which may be more difficult for a patient to evacuate (Figure 1) [9]. Water and solute secretion into the intestinal lumen are essential for lubrication and influence stool consistency [10]. Fluid secretion into the gastrointestinal tract is in part regulated by guanylate cyclase-C (GC-C) [10]. Patients with constipation may have impaired or decreased expression of GC-C compared with an unaffected cohort [40]. In contrast, increased GC-C expression may lead to diarrhea [10]. Accordingly, the GC-C receptor is a pharmacologic target for medications, such as plecanatide (currently only approved for use in the US) and linaclotide, that activate this system and thereby enhance fluid secretion into the gut (Figure 3) [24,41]. Although otherwise associated with few adverse events in adults, these agents are associated with an increase in diarrhea likely related to the mechanism of action [24,41]. Different rates of diarrhea between the two may be attributable to pH-independence and affinity to the GC-C receptor [34].

Gut motility and contractility play an important role in stool transit. Small non-peristaltic contractions aid in gut absorption of water [9]. Fiber supplements have been shown to cause mechanical stimulation of the gut mucosa, causing softer stools and faster colonic transit [43]. Larger peristaltic contractions (also called high-amplitude propagated contractions [HAPCs]) help propel stools along the colonic lumen [9]. A decrease in frequency of these larger contractions may be one pathophysiological mechanism involved in constipation [9].

As noted, behavioral and psychological factors can cause or contribute to constipation. Constipation may start in childhood, when most cases are considered idiopathic [44]. Withholding of stools after a difficult bowel movement, a common etiology of functional constipation in the pediatric population, leads to water absorption from the fecal mass, increasing evacuation difficulty, rectal distension, loss of rectal sensation, and eventually loss of the normal defecatory urge [44]. After children with constipation enter adulthood, one-fourth of them may continue to experience symptoms [45]. Psychological factors, such as anxiety and stress, may contribute to constipation by increasing skeletal muscle tension, which can lead to dyssynergic defecation [46].

Alterations in the gut microbiota may also affect gut motility [22]. Various gases are produced by gut microorganisms, including hydrogen sulfide, carbon dioxide, and methane [22]. The increased production of hydrogen and carbon dioxide from bacterial fermentation of oligosaccharides may cause symptoms of bloating [22]. The increased production of methane in the gut is thought to slow gut motility, contributing to constipation in some patients [22]. Patients with constipation have decreased levels of Bifidobacteria, Lactobacillus, Clostridium leptum, and Faecalibacterium prausnitzii and increased levels of Bacteroides spp and Enterobacteriaceae [47].

Functional and physiological abnormalities of the anorectum can additionally be involved in contributing to constipation [9,46]. Dyssynergia, likely the most common DD, is a spectrum of dysfunctional or disordered contractions and relaxations in muscles involved in defecation. Dyssynergia may present with pathologic habitual behaviors, such as avoidance of defecation (often due to a painful anal fissure), along with various other comorbidities, including back injury, brain–gut dysfunction, eating disorders, and a history of sexual or physical abuse [9]. Less common causes of DD include mechanical factors, such as rectal intussusception, prolapse, rectocele, and abnormal perineal descent [9]. Injuries to pelvic floor muscles during childbirth are attributable to higher rates of constipation in women [7]. When diagnosed in the primary care setting, gynecological referral may be warranted. Guidelines for managing constipation due to defecatory disorders favor pelvic floor retraining by biofeedback as opposed to laxatives [28].

Bile acids may have laxative effects and, as a result, impaired bile acid synthesis can also contribute to constipation [22]. Bile acids inhibit apical Cl^−^/OH^−^ exchange, increase permeability of the intestinal mucosa, activate intracellular secretory mechanisms, and are beneficial to propulsive colonic contractions, thereby improving colonic motility and evacuation [22]. In a study of IBS, the constipation-predominant group had the lowest bile acid values and significantly decreased percentages of two of the most potent secretory bile acids [48].

Medications may affect water regulation in the gut or gut motility. Medications that can cause constipation are myriad, including antidepressants—notably tricyclic antidepressants—that exert profound anticholinergic effects as well as affect serotonin levels [1,9,49]. Serotonin is known to be involved in the regulation of gastrointestinal motility [22]. Antihypertensive drugs, such as certain calcium channel antagonists, may inhibit smooth muscle contraction in the intestinal tract [1,9]. As a result, medications in this class may contribute to increasing colonic transit time. Analgesics, especially opiates, contribute to constipation [1,9]. Opioid receptors are located throughout the gut [50], but constipation is largely caused by delayed transit in the colon and increased colonic fluid absorption resulting in harder, firmer stools [51]. Oral iron supplements are also classically associated with constipation [1,9].

Certain systemic diseases are associated with constipation. Neurological disorders that may induce constipation include autonomic neuropathy, Parkinson’s disease, multiple sclerosis, and certain spinal cord injuries [1,9]. Neurological causes of constipation are complex, as they can include neural dysfunction and systemic factors, such as impaired mobility [9]. If the neural connection between the brain and the gut is affected, this may alter bowel function. Endocrine or metabolic disorders may contribute as well, including diabetes, hypothyroidism, and hypercalcemia [1]. These conditions can impact gut function and motility. As a case in point, constipation may affect up to 60% of patients with diabetes [52] (a common disease), helping to explain the frequency of such complaints in the general patient population. Although hypothyroidism may cause constipation, this is not a common condition among patients presenting with constipation [53]. Mechanical bowel obstruction of any etiology can also cause constipation; however, obstructions of acute onset may present differently than those of gradual or subtle onsets [1]. These conditions should always be among the first diagnoses to be excluded prior to continuing the patient’s workup and further evaluation.

Pathophysiological factors often overlap and interact in cases of chronic constipation [1]. Researchers seeking to identify an integrated explanatory model for IBS identified three main components that may be associated with constipation: alterations in the peripheral regulation of gut function (sensory and secretory mechanisms), psychological distress, and brain-gut signaling (visceral hypersensitivity) [54]. As discussed above, sensory and secretory mechanisms of constipation can be impacted by diet (e.g., liquid intake, fiber), the gut microbiome, anorectal abnormalities, and bile acid composition. Depression and anxiety as well as somatization and psychotic disorders were significantly higher (*p* < 0.05) in patients with constipation compared with controls; these types of psychological stressors positively correlated with constipation symptoms (e.g., straining, sensation of anal blockage) [55]. There is evidence suggesting differences in brain-gut signaling in patients with constipation, and such patients often have a higher threshold to sense the urge to evacuate [54].

## 5. Case Studies

### 5.1. Case Study 1

A 40-year-old woman reports an average of 2 spontaneous bowel movements each week for the past 6 months. She describes her bowel movements as rarely resulting in a sensation of complete evacuation. She has been researching her symptoms on the internet and has tried to increase her exercise and has purchased fiber supplements from her supermarket. After several weeks she had not observed any improvements in her constipation.

She consulted her local pharmacist, who encouraged her to use a different brand of fiber supplement, but this also did not improve her symptoms. As a result, the pharmacist suggested she try a laxative. The first recommended laxative also failed to relieve the symptoms, so the pharmacist recommended an alternative laxative. During this time, she felt her symptoms were beginning to worsen and she started experiencing abdominal pain and bloating. Her stools also moved from being lumpy and sausage-like (BSFS type 2) to hard, separate lumps (BSFS type 1).

She is now seeking guidance from her PCP to address her abdominal pain, which is her primary symptomatic concern. She has not been diagnosed with depression or anxiety but feels that her constipation and abdominal pain are negatively affecting her QoL. She is otherwise healthy and has not been prescribed any medication that is typically associated with secondary constipation.

As this patient does not have alarm symptoms and secondary constipation is unlikely, her PCP made the clinical diagnosis of IBS-C and continued managing her care. A review of prior treatments determined inadequate trials of OTC laxatives. The patient had previously tried MiraLAX (a polyethylene glycol-based osmotic laxative), and experienced modest but only intermittent relief of constipation and abdominal discomfort. Her PCP began an appropriate treatment course that included use of lubiprostone, which was associated with initial improvement, but was discontinued due to diarrhea and intolerable nausea despite instructions on the optimal manner in which to use the medication. In consultation with fellow internists and a gastroenterology colleague, the patient’s primary physician started her on plecanatide 3 mg daily, which began to have an effect over the next several days, with the patient reporting marked improvement in both abdominal discomfort and constipation within 6 days of treatment. After 2 weeks, the patient is nearly symptom-free, and now experiences well-formed bowel movements up to 3 to 4 times weekly.

### 5.2. Case Study 2

A 53-year-old woman reports an average of one complete spontaneous bowel movement each week for the past several years, which has been stable in nature. She describes most of her bowel movements as incomplete evacuations. She also notes abdominal discomfort and pain, which typically precede defecation and are generally relieved by passage of a bowel movement. Of note, there are no alarm signs/symptoms or so-called “red flags”, including the absence of rectal bleeding and unintentional weight loss, and there is no consistent or concerning change in stool size/caliber. She has not undergone prior colonoscopy or other colorectal cancer–screening modalities.

Increased liquid intake, dietary and supplemental fiber (such as psyllium), and aerobic exercise have been of only modest benefit. The use of various OTC stool softeners and laxatives, along with enemas, has provided intermittent but generally short-lived improvement in her symptoms. Her stools have consistently ranged from BSFS type 1–3, without a consistent pattern.

She now seeks advice from her PCP to address her abdominal pain and constipation, her two main symptomatic issues. There are no known alarm signs or symptoms, as noted above. She has no other organic pathology that is likely to contribute, including the absence of other underlying comorbid conditions. She does not use any medications (prescription or OTC) typically associated with secondary constipation/alterations in GI tract motility. However, with worsening constipation and no history of prior colonoscopy, the patient is referred to a gastroenterologist for colorectal cancer screening.

### 5.3. Case Study 3

A 23-year-old man presents to his PCP with 6 months of altered bowel habits. He reports 2 to 3 bowel movements per week associated with significant straining and incomplete evacuation. He underwent abdominal surgery the previous year for an episode of acute appendicitis with an uncomplicated laparoscopic appendectomy. Since then, he reports intermittent use of tramadol 50 mg every other day. He has no abdominal pain and no “red flag” or alarm symptoms (with the absence of hematochezia, unintentional weight lost, or change in stool caliber). He also reports a longstanding history of major depressive disorder and has been using paroxetine 20 mg daily for 3 years. He currently reports feeling well overall, with a recent Patient Health Questionnaire-9 (PHQ-9) score of 3. He has been taking a daily OTC senna-based laxative without effect.

Abdominal examination is non-tender and non-distended with old, well-healed surgical scars present. Rectal examination is notable for normal rectal tone, no masses, and hard stool in the rectal vault. All blood tests are within the normal range, including a complete blood count, metabolic panel, and thyroid-stimulating hormone (TSH) level.

In consultation with a pain management specialist, the patient is able to be weaned off the tramadol and started on non-opiate analgesia with good effect. In consultation with the patient’s psychiatrist, the patient was switched to a tricyclic antidepressant. The PCP then recommended the patient initiate MiraLAX 17 g daily with change in bowel habits to 2 bowel movements (BSFS type 3–4) every day.

### 5.4. Case Study 4

A 32-year-old female has had 2 vaginal deliveries, one requiring forceps, and both resulting in second-degree vaginal tears. She reports an average of 2 to 3 bowel movements weekly (BSFS type 1–2) since the birth of her second child approximately 1 year ago. She complains of straining, requiring that she spend 15–20 min on the toilet in order to pass a complete bowel movement. She requires digital maneuvers to initiate and complete a bowel movement.

She has tried supplemental fiber in the past, but this was discontinued due to increased bloating and gas. She was managing reasonably well with the occasional use of OTC laxative preparations, but recently, due to rectal pain associated with her bowel movements, the laxatives caused excessive discomfort with the increasing frequency with which she now needs to use the toilet. She is now seeking evaluation by her PCP.

Her PCP performed a digital rectal exam, which revealed suspected DD, and checked her TSH and hemoglobin (Hgb); both were within normal range. The patient was referred for gastroenterology subspecialty consultation and formal anorectal evaluation, which confirmed diagnosis of DD. Pelvic floor exercise and biofeedback were prescribed, ultimately improving the patient’s symptoms.

## 6. Summary

The causes of constipation are multifactorial and complex; however, constipation can be diagnosed and managed in the primary care setting. It is important for primary care providers (including internists, gynecologists, physician assistants, and nurse practitioners) to be familiar with the different subtypes of chronic constipation in order to diagnose and hence treat each patient accordingly. PCPs should also be familiar with alarm signs and symptoms that should trigger referral (or expedited referral) to a gastroenterologist or other appropriate specialist as needed.

## Figures and Tables

**Table 1:** Comparison between symptoms of IBS-C and CIC.

Condition	Rome IV Criteria for Diagnosis	Other Considerations
IBS-C	Recurrent abdominal pain (≥1 per week Change in stool frequency Change in form of stool ≥ 25% of bowels movements are BSFS type 1 or 2 < 25% of bowel movements are BSFS type 6 or 7 [4,10,22]	Patients must present with abdominal pain for a diagnosis of IBS [2] Abdominal bloating is often present, though not required for diagnosis [2] Abdominal pain or discomfort may be relieved with defecation [23,24]
CIC	Patients should meet ≥2 of the following in the last 3 months [4,10] < 3 bowel movements/week Straining for > 25% of bowel movements Lumpy or hard stools (BSFS type 1 or 2) for > 25% of bowel movements Sensation of incomplete defecation in > 25% of bowel movements Sensation of anorectal obstruction/blockage in > 25% of bowel movements Manual maneuvers to facilitate > 25% of bowel movements Patients do not meet the Rome IV criteria for IBS-C	Patients who have some similar symptoms to IBS-C but who do not meet IBS-C criteria are diagnosed with CIC [4,10] CIC is commonly determined by the frequency of bowel movements [3] Patients may experience bloating, abdominal pain, or discomfort, but these are not considered as main symptoms for CIC [4,9,10]

BSFS, Bristol Stool Form Scale; CIC, chronic idiopathic constipation; IBS-C, irritable bowel syndrome with constipation.

**Table 2:** Causes of secondary constipation [1].

Secondary Constipation Subtype	Possible Underlying Causes
Medications	Iron supplements, calcium supplements, antidepressants, antihypertensive drugs, opioids, antihistamines, anticholinergics, etc.
Neurological, endocrine, and metabolic	Autonomic neuropathy, Parkinson’s disease, multiple sclerosis, spina bifida, spinal cord injuries, diabetes, hypothyroidism, hypercalcemia, pregnancy
Bowel obstruction	Obstructing colonic cancer, luminal stenosis, abdominal adhesions, etc.

## Data Availability

No new data were created or analyzed in this study. Data sharing is not applicable to this article.
